# Lipid Nanoparticles for Enhancing the Physicochemical Stability and Topical Skin Delivery of Orobol

**DOI:** 10.3390/pharmaceutics12090845

**Published:** 2020-09-03

**Authors:** Min-Hwan Kim, Yae-Eun Jeon, Soobeen Kang, Jae-Young Lee, Ki Won Lee, Ki-Taek Kim, Dae-Duk Kim

**Affiliations:** 1 College of Pharmacy and Research Institute of Pharmaceutical Sciences, Seoul National University, Seoul 08826, Korea; mhkim305@snu.ac.kr (M.-H.K.); ayejeon@snu.ac.kr (Y.-E.J.); 2 Department of Material Science and Engineering, Seoul National University, Seoul 08826, Korea; kang9997@snu.ac.kr; 3 College of Pharmacy, Chungnam National University, Daejeon 34134, Korea; jaeyoung@cnu.ac.kr; 4 Department of Agricultural Biotechnology, Seoul National University, Seoul 08826, Korea; kiwon@snu.ac.kr; 5 R&D Center, BOBSNU, Suwon 16229, Korea; 6 Department of Biomedicine, Health & Life Convergence Sciences (BK21 Four), College of Pharmacy and Natural Medicine Research Institute, Mokpo National University, Jeonnam 58554, Korea

**Keywords:** orobol, nanostructured lipid carrier (NLC), physicochemical stability, Strat-M membrane, human cadaver skin, topical skin delivery, skin irritation

## Abstract

Orobol is one of the major soy isoflavones, and has been reported to have various pharmacological activities, including an anti-skin-aging effect. However, since it has low solubility in water and physicochemical instability, the formulation of orobol for delivery into the dermal layer of the skin could be challenging. The objective of this study was to prepare lipid nanoparticles formulations of orobol to enhance its stability as well as its deposition into the skin. Formulations of orobol-loaded solid lipid nanoparticles (SLNs) and nanostructured lipid carriers (NLCs) were characterized in terms of their mean particle size, entrapment efficiency, and morphology. The nano-sized spherical NLCs formulations maintained the stability of orobol for up to 28 days. Moreover, the NLCs formulation significantly increased the in vitro deposition of orobol into both Strat-M membranes and human cadaver skin compared with the other formulations. Additionally, the NLCs formulation did not cause significant skin irritation in clinical study. These results demonstrate that a shea butter-based NLC formulation could be a promising and safe carrier system for improving the stability of orobol and enhancing its topical skin delivery.

## 1. Introduction

Long-term ultraviolet (UV) exposure is one of the major causes of photoaging of the skin, which results in irreversible and progressive structural changes of the skin [1]. The clinical signs of photoaging include wrinkles, skin roughness, and decreased skin elasticity. Studies have shown that, when exposed to UV light, reactive oxygen species (ROS) are generated in the skin, which then activate c-Jun N-terminal kinase and stimulate the expression of matrix metalloproteinase-1 (MMP-1), thereby up-regulating the degradation of collagen, elastin, and hyaluronic acid in the dermis of the skin [2,3,4].

Isoflavones derived from soy beans have been widely used as potential anti-skin-aging compounds [5,6]. Numerous studies have reported that genistein, a major soy isoflavone, could suppress the generation of ROS and expression of MMP-1 and inducible nitrogen oxide synthase (iNOS), thereby exerting anti-wrinkle, skin-protective, and hydration effects against UV-induced photoaging [7,8]. However, due to its low skin permeability as well as low aqueous solubility, skin accumulation of genistein (NaCl solution; 140 mM) was only 0.1 μM per g of skin [9]. Thus, nano-carrier systems such as microemulsions, liposomes, and lipid nanoparticles have been exploited to enhance topical skin delivery of genistein and reach its therapeutic concentration in target cells of skin [9,10,11].

Orobol (5,7,3′,4′-tetrahydroxyisoflavone), a metabolite form of genistein, is one of the naturally occurring soy isoflavones from plants. In nature, it exists in fermented soybean, or else it is produced by liver microsomes after the ingestion of soybean [12]. Recently, it was reported that orobol has various pharmacological activities, including neuronal-protective, anti-Alzheimer, anti-obesity, and anti-proliferative effects [12,13,14]. Moreover, orobol shows a potent anti-photoaging effect by suppressing UV-induced MMP-1 expression in human keratinocyte (HaCaT) cells and human dermal fibroblasts (HDFs) at a concentration of 1 μM, whereas the same concentration of genistein do not exert significant suppression of MMP-1 expression, indicating that orobol can be a next-generation anti-skin-aging ingredient superior to genistein [15]. However, similar with the limits of genistein, formulation of orobol for absorption through the stratum corneum (SC) of skin and for reaching the dermal layer is challenging due to its low aqueous solubility (44.9 μg/mL; Table 1) [16]. Moreover, it is unstable when exposed to sunlight, and its color is changed to yellow in various vehicles (Supplementary Figure S1). Thus, a proper formulation is required for the topical skin delivery of orobol in order to exert its anti-skin-aging effect. Nonetheless, studies on its topical application and formulation study for skin delivery have not been reported yet.

Among various nano-carrier systems, solid lipid nanoparticles (SLNs) and nanostructured lipid carriers (NLCs) have been considered as promising formulations for the topical or transdermal application of poorly water-soluble and/or instable compounds [17,18]. SLNs consists of a solid lipid as the inner phase, and are stabilized by various surfactants. It is known that SLNs can enhance the stability of compounds encapsulated in the inner phase and can control the release of compounds [19]. When they are applied on the skin, they can not only provide a skin hydration effect, but can also enhance the penetration through the SC, since the surfactant(s) act as a permeation enhancer [20]. NLCs, also known as second-generation of SLNs, can be prepared by using a mixture of “solid” lipids and “liquid” oils as the inner phase. Thus, NLCs show higher loading capacity and physicochemical stability during storage compared to SLNs [21]. Therefore, SLNs and NLCs could be suitable nano-carrier systems for overcoming the physicochemical drawbacks of orobol, such as its poor water-solubility and photoinstability.

**Figure 1:**
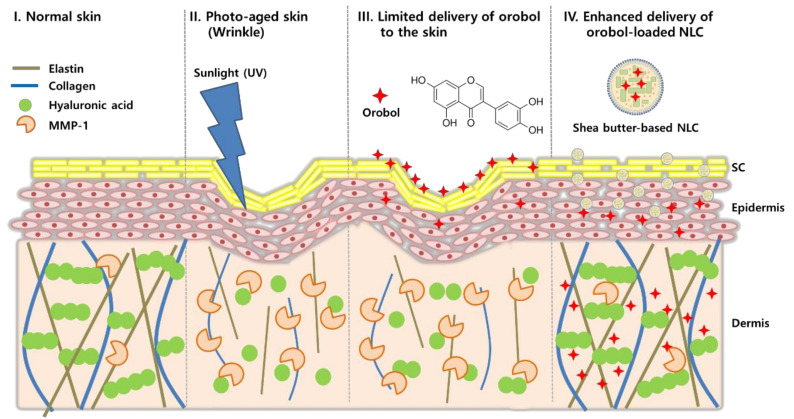
Scheme of orobol-loaded nanostructured lipid carriers (NLCs) to enhance photostability and topical skin delivery.

The objective of this study was to investigate SLNs and NLCs formulations of orobol for topical skin delivery, thereby enhancing its photostability and deposition into the skin (Figure 1). To our knowledge, this is the first study to report skin absorption of orobol and its lipid nanoparticles. Various orobol-loaded SLNs and NLCs were prepared and characterized in terms of their mean particle size, size distribution, entrapment efficiency (EE), drug content, and morphology. Also, their photostability was investigated by observing changes in particle size, content, and color. Then, in vitro skin deposition was assessed using artificial skin membranes (Strat-M^TM^) and human cadaver skin. In addition, skin irritation was also investigated after topical application of the final formulations to human.

## 2. Materials and Methods

### 2.1. Materials

Orobol (purity ≥ 98.0%) was provided from Wuhan ChemFaces Biochemical Co., Ltd. (Wuhan, China). Medium-chain glycerides (MCTs), refined cocoa butter, and shea butter were obtained from DAMY Chemical Co., Ltd. (Seoul, Korea). Capmul MCM EP was gifted by ABITEC Co. (Peterborough, UK). Labrafac CC, Labrasol (PEG-8 caprylic/capric glycerides), and Transcutol HP were gifted by Gattefossé Co. (Saint Priest, France). Miglyol was purchased from IOI Oleo GmbH (Hamburg, Germany). Tween 20 and sodium dodecyl sulfate (SDS) were obtained from Sigma-Aldrich Chemical Co. (St. Louis, MO, USA). Phosphate-buffered saline (PBS) was purchased from Lonza, Ltd. (Basel, Switzerland). Strat-M^TM^ membranes (2.5 cm in diameter) were purchased from Merck Millipore (Billerica, MA, USA). High-performance liquid chromatography (HPLC)- grade methanol, acetonitrile, and water were purchased from Thermo Fisher Scientific Co. (Pittsburgh, PA, USA). Female human cadaver skin (2 × 2 cm^2^) was purchased from a skin and tissue bank (Hans Biomed Co., Seoul, Korea). Studies that use human cadaver skin from a tissue bank do not require approval from the Institutional Review Board (IRB) or ethics committee (Article 33, Enforcement Rule of the Bioethics and Safety Act in the Republic of Korea) [22].

### 2.2. Preparation of Orobol-Loaded Formulations

#### 2.2.1. Solubility Study

The solubility of orobol was assessed in various vehicles, including deionized water (DW), oils, and surfactants, by placing an excess amount of orobol into a tube containing 1 mL of each vehicle. The mixtures were shaken for 72 h at 25 °C in a vortex shaker (Vortex-Genie 2; Scientific Industries, Inc., Bohemia, NY, USA) at 50 rpm to reach a saturated state. Then, the samples were centrifuged at 16,000× *g* for 5 min. The supernatant was filtered through a 0.2 μm syringe filter to remove undissolved orobol. The filtered samples were appropriate diluted with methanol, and then the concentration of orobol was analyzed by liquid chromatography with tandem mass spectrometry (LC-MS/MS).

#### 2.2.2. Preparation of Orobol-Loaded NLCs and SLNs

NLCs and SLNs were prepared by using cocoa butter or shea butter as a solid lipid phase. Based on the results of the solubility study (Table 1), Transcutol and Capmul MCM EP was selected as a surfactant phase and an oil phase of NLCs, respectively. The composition of the prepared NLCs (F2 and F4) is shown in Table 2, while SLNs without oil (F1 and F3) were prepared for comparison. Hot homogenization followed by sonification method was adapted to prepare orobol-loaded NLCs and SLNs, based on previous reports with slight modification [23,24]. Briefly, cocoa butter or shea butter was melted at 70 °C. In a separate tube, an exact amount of orobol (0.05%, *w*/*w*) was mixed with transcutol and oil (Capmul MCM EP), after which was then added into the melted solid lipid phase. DW containing tween 20 was added dropwise into the above mixture, followed by applying tip-sonication (Vibra-Cell; Sonics & Materials, Inc., Newton, CT, USA) at 26% amplitude, pulsed on for 2 s and off for 3 s. Then, the mixture was filtered using a 0.45 μm syringe filter (Watman; Ministat RC15, Sartorius, Germany) to remove the unentrapped orobol and residual excipients that were not included in the nanoparticles. The filtrates were cooled at room temperature to obtain NLCs in aqueous solution, which were then used for further experiments. For the preparation of orobol-loaded SLNs, the above-mentioned method, except for the process of mixing with oil (Capmul MCM EP), was adapted.

### 2.3. Characterization of Orobol-Loaded Formulations

#### 2.3.1. Mean Particle Size and Size Distribution

The mean particle size, intensity distribution of particle size, and polydispersity index (PDI) of the orobol-loaded NLCs and SLNs in aqueous solution were determined by using an electrophoretic light-scattering (ELS) spectrophotometer (ELS 8000; Otsuka Electronics Co., Ltd., Tokyo, Japan). All measurements were performed in a quartz cuvette and in triplicate at 25 °C.

#### 2.3.2. Particle Morphology

The morphologies of the orobol-loaded SLNs and NLCs particles were observed by energy-filtering transmission electron microscopy (TEM; LIBRA 120; Carl Zeiss, Jena, Germany) at 80 kV. The samples (5 μL) were dropped on a copper grid and were negatively stained with 2% uranyl acetate for 10 s. The copper grid with the samples was washed twice with DW, followed by drying at room temperature prior to the operation.

#### 2.3.3. Entrapment Efficiency (EE) and Content of Orobol

The EE of orobol in the SLNs and NLCs formulations was determined by using a previous method with slight modification [25]. Briefly, the amount of orobol in the SLNs and NLCs aqueous solutions (*A_fitrate_*) was determined after completely dissolving them by adding methanol, followed by vortex shaking. The amount of unloaded orobol in the filtrate solution (*A_free_*) was separately determined after ultrafiltration using Amicon Ultra-4 (Millipore, Country Cork, Ireland) with a 10 kDa molecular weight cut-off. Then, the concentration of orobol was analyzed by LC-MS/MS after appropriate dilution with methanol. The EE was calculated from the following Equation (1):

(1) $$\mathrm{Entrapment}\;\mathrm{efficiency}\;\left(EE;\;\%\right)=\frac{A_{fitrate}-A_{free}}{A_{total}}\times 100$$

where *A_total_* is the theoretical amount of orobol in the formulations, *A_fitrate_* is the amount of orobol in the SLNs and NLCs aqueous solutions, and *A_free_* is the amount of free orobol in the aqueous phase of the formulations.

In order to determine the content of orobol in the SLNs and NLCs formulations, the total weight of each formulation (*W_total_*) was measured after lyophilization. Then, the orobol content of the formulations was calculated from the following Equation (2):

(2) $$\mathrm{Loading}\;\mathrm{content}\;\left(\%\right)\;=\;\frac{A_{actual}}{W_{total}\;-\;A_{free}}\;\times \;100$$

where *W_total_* is the total weight of the lyophilized formulation.

#### 2.3.4. Powder X-ray Diffraction (pXRD) and Attenuated Total Reflectance-Fourier Transform Infrared (ATR-FTIR) Study

To determine the crystallinity of orobol, pXRD patterns were measured by using Bruker D8 Advance diffractometry (Bruker Co., Karlsruhe, Germany) with a Cu-Kα source at 40 kV of voltage and 40 mA of current. Each sample (i.e., orobol, solid lipids, physical mixtures (PMs) of F1 and F3, and lyophilized F1 to F4) was scanned in the 2θ range of 3° to 40°, where the scanning was set at 1°/min. In order to figure out the molecular interactions in the SLNs and NLCs, ATR-FTIR spectroscopy (JASCO FT/IR 4200; JASCO Company Ltd., Hachioji, Japan) was performed. The FTIR spectra of the orobol, solid lipids, PMs of F1 and F3, and lyophilized F1 to F4 were recorded over a scanning range of 600–4000 cm^−1^ with a resolution of 4.0 cm^−1^.

### 2.4. Stability Study

The stability of orobol in the SLNs and NLCs formulations was observed by determining the change of mean particle size and the amount of orobol in the aqueous solutions. Samples were stored at room temperature under sunlight. At each time point (0, 3, 7, 14, 21 and 28 days), the mean particle size of the samples was measured by using a spectrophotometer (ELS 8000). The content of orobol in the SLNs and NLCs was determined after completely dissolving the formulations with methanol. The amount of orobol released in the aqueous solutions was separately determined after ultrafiltration as described above, and then was subtracted from the content. Changes in orobol content in the SLNs and NLCs was represented as a percentage of the initial content (0 days). Color changes in the orobol-loaded formulations (F1 to F4) and orobol (0.05%, *w*/*w*) in various vehicles (i.e., DW, transcutol, ethanol, and Capmul MCM EP) were also observe for 14 days with or without exposure to sunlight.

### 2.5. In Vitro Deposition Study Using Artificial Membrane and Human Cadaver Skin

In vitro artificial membranes (Strat-M; Merck Millipore) deposition of orobol was evaluated by using Keshary–Chien diffusion cells (surface area of 1.77 cm^2^) at 32 °C [16]. Strat-M membranes were placed between the donor and receptor cells, laying the shiny side upward. The receptor solution was PBS containing SDS (0.5%, *w*/*v*) to maintain sink condition and was constantly stirred at 600 rpm. Various orobol-loaded SLNs and NLCs formulations (F1 to F4) or an orobol suspension in DW was applied to the donor cells at a concentration of 0.5 mg/mL of orobol (1.0 mL) and sealed with parafilm to prevent evaporation. After applying the samples for 3 h and 6 h, the Strat-M membranes were separated from the diffusion cells, after which were washed five times with methanol [26]. They were then collected into a 2.0 mL tube, and orobol was extracted from the Strat-M membranes with a mixture of acetone and methanol (70:30, *v*/*v*, 1.5 mL). Tubes were shaken for 3 h, followed by centrifugation for 5 min at 16,000× *g*. The supernatant was transferred into a vial, and was then appropriately diluted with methanol for LC-MS/MS analysis.

In vitro female human cadaver skin deposition of orobol was evaluated using the same diffusion cells as described above, except that the human cadaver skin was placed between the diffusion cells, facing the SC side upward. After applying the samples for 3 h and 6 h, the skin was removed from the diffusion cells, and was then washed via the above-mentioned method. Then, the skin was chopped with scissors and placed in a mortar, which was then ground using a pestle with the addition of liquid nitrogen [16]. The skin powder was collected into a 2.0 mL tube, and methanol (1.5 mL) was added. The tube was shaken for 3 h to extract orobol from the skin, and then centrifuged at 16,000× *g* for 5 min. The supernatant was appropriately diluted with methanol, and injected into the LC-MS/MS system. The deposited amount of orobol (ng/cm^2^) at 3 h and 6 h was normalized by the surface area of the diffusion cells.

### 2.6. LC-MS/MS Analysis of Orobol

The orobol amount in samples was determined using an Agilent LC-MS/MS system (Agilent Technologies, Santa Clara, CA, USA) which is equipped with an Agilent Technologies 1260 Infinity HPLC system and an Agilent Technologies 6430 Triple Quad LC-MS system. Aliquot (20 μL) of the samples was injected into a Kinetex C_18_ column (2.6 μm, 100 mm × 4.6 mm; Phenomenex, Torrance, CA, USA). The mobile phase consisted of acetonitrile and water containing 0.2% formic acid (15:85, *v*/*v*) at the flow rate of 0.3 mL/min. Orobol was analyzed in multiple reaction-monitoring (MRM) mode with positive electrospray ionization (ESI). Parameters of the ESI were as follows: Gas flow, 9 L/min; gas temperature, 300 °C; capillary voltage, 4000 V; and nebulizer pressure, 25 psi. The *m*/*z* value of the precursor to product ion of orobol was 287.2 to 153.0. The collision energy, fragment voltage, and cell accelerator voltage for the MRM mode were 30 eV, 165 V, and 4 V, respectively. The retention time of orobol was 3.06 min. The analytical data were processed using MassHunter Workstation Software Quantitative Analysis (vB.05.00; Agilent Technologies). The signal-to-noise ratio on the lower limit of quantification (10.0 ng/mL) was higher than 5.0, and there was no interference from any other substance (Supplementary Figure S2). The calibration standard curve was obtained by serial dilution of stock solution (1.0 mg/mL orobol in methanol), which had a final concentration range of 10.0–1000 ng/mL. The peak area of orobol was linear within the concentration range with higher than 0.998 of the correlation coefficient (*r^2^*) (Supplementary Figure S2).

### 2.7. Skin Irritation Study in Humans

The skin irritation study of orobol-loaded NLCs (F4) and blank NLCs (F4 without orobol) formulations in aqueous solution was performed in 30 healthy Korean female volunteers (age 20–55). The experimental procedures were approved by the Institutional Review Board (KDRI-IRB-17318) of the Korean Dermatology Research Institute (Seongnam, Korea). IRB proposal was submitted on 15 August 2017, and was approved on 14 November 2017. Briefly, the application site (i.e., back) of each subject was washed with 70% ethanol prior to applying the samples. Then, a 15 μL aliquot of each sample was applied on the back of the subject, and sealed with a medical plaster. After application for 24 h, the plasters were removed, and the skin irritation score was measured three times (30 min, 24 h, and 48 h after washing) by following the guidelines of the International Contact Dermatitis Research Group (ICDRG) and the Personal Care Products Council (PCPC) (Supplementary Table S1). The, the skin irritation index was calculated to assess the irritation of the formulations on the skin based on the standard operating procedure of the dermal classification system by the Environmental Protection Agency (EPA) (Supplementary Table S2). Skin irritation index was calculated from the following Equation (3):

(3) $$\mathrm{Skin}\;\mathrm{irritation}\;\mathrm{index}\;\;=\;\frac{\sum \;\mathrm{Irritation}\;\mathrm{score}\;\mathrm{at}\;30\;\min,\;24\;h\;\mathrm{and}\;48\;h}{\mathrm{Total}\;\mathrm{number}\;\mathrm{of}\;\mathrm{subjects}}$$

### 2.8. Statistical Analysis

All experiments were conducted at least three times, after which the data are expressed as the mean ± standard deviation (S.D). Analysis of variance (ANOVA) with Tukey’s post-hoc test (IBM SPSS Statistics Software, v 21.0; IBM Co., Armonk, NY, USA, 2012) were used for statistical analysis. A *p*-value of less than 0.05 was considered statistically significant.

## 3. Results

### 3.1. Preparation of Orobol-Loaded NLCs and SLNs

Table 1 shows the solubility of orobol in various oils and surfactants, together with that in water (44.9 μg/mL). The solubility of orobol was the highest in Capmul MCM EP (12.4 ± 0.2 mg/mL) among the oil phases, followed by Miglyol, MCT and Labrafac CC. Among the surfactants, the solubility of orobol was the highest in Transcutol (67.9 ± 2.0 mg/mL), followed by Labrasol and Tween 20. Thus, Transcutol and Capmul MCM EP were selected as the surfactant and as the oil, respectively, for the formulation of SLNs and NLCs. Cocoa butter and shea butter were selected as the solid lipids for the inner phase. Table 2 summarizes the compositions of the orobol-loaded SLNs (F1 and F3) and NLCs (F2 and F4) formulations. Tween 20 was chosen as a surfactant for reducing the surface tension of the lipid nanoparticles.

### 3.2. Characterization of Orobol-Loaded SLNs and NLCs

**Figure 2:**
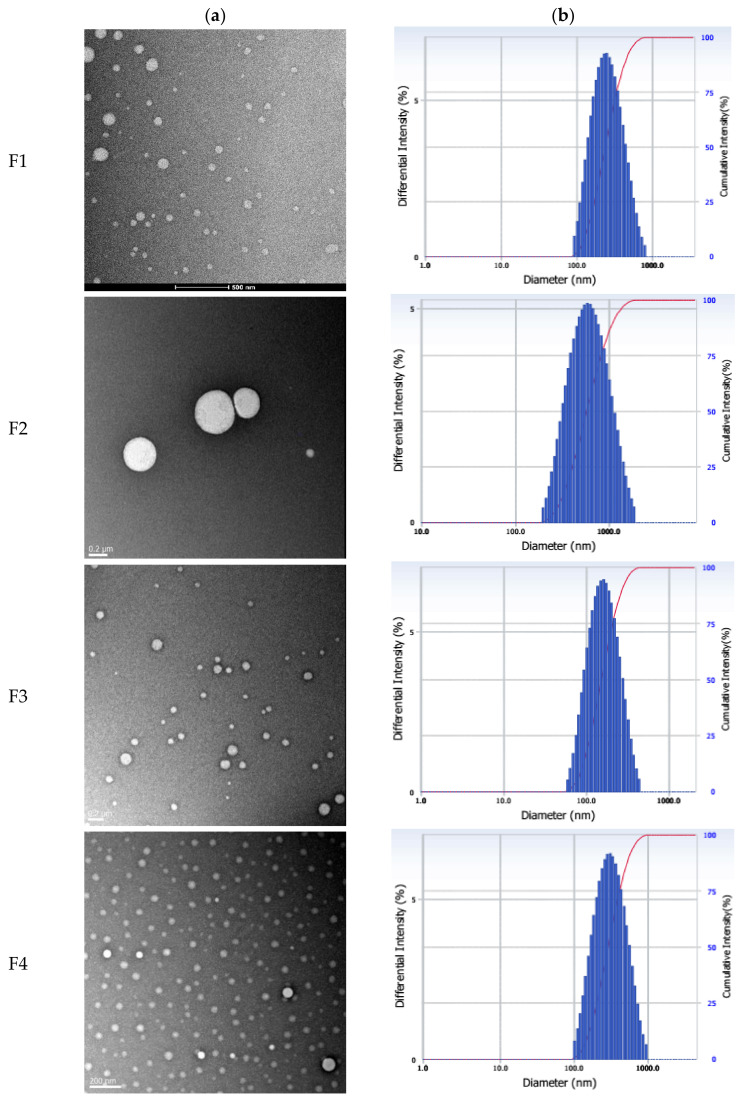
(**a**) Morphological shapes of the orobol-loaded formulations (F1 to F4) observed by transmission electron microscopy (TEM) and (**b**) their particle size distribution. The scale bars represent 0.2 μm.

The mean particle size, PDI, EE, and drug content of the orobol-loaded formulations are presented in Table 3. The TEM images and particle size distribution of orobol-loaded formulations are shown in Figure 2. Nano-sized particles (133–165 nm) were observed in the prepared orobol-loaded SLNs. The mean particle sizes of orobol-loaded NLCs where the oil was added into the solid lipid core increased up to 246 and 498 nm. However, all formulations showed a narrow size distribution. The formation of spherical nano-sized particles in all formulations was confirmed via TEM. Moreover, the diameter of the prepared formulations in the TEM images was consistent with the mean particle size obtained by using an ELS spectrophotometer. The EE and content of the orobol-loaded SLNs and NLCs ranged from 95.7% to 97.2% and from 0.91% to 0.97%, respectively.

**Figure 3:**
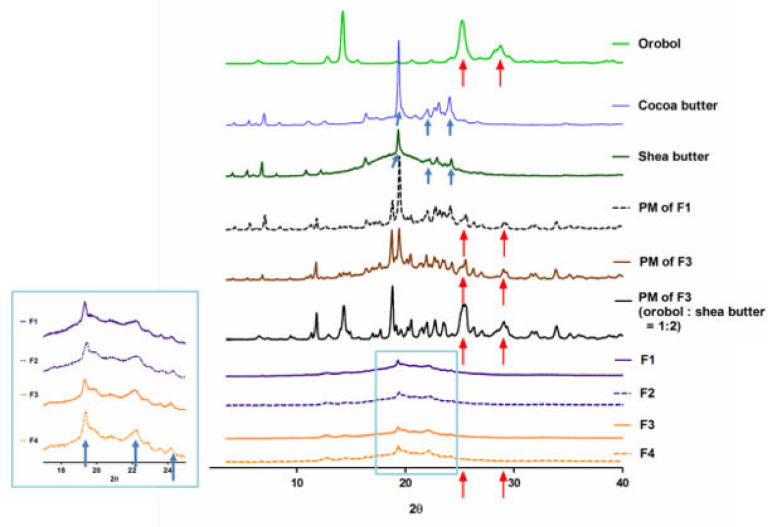
X-ray diffraction patterns (relative intensity) of the orobol, solid lipids (i.e., cocoa butter and shea butter), physical mixtures (PMs) of F1 and F3, and lyophilized SLNs and NLCs formulations (F1 to F4). Crystalline peaks of the orobol are presented at 25.5° and 28.8° 2θ (red arrows), while those of the lipids are presented at 19.3°, 22.1°, and 24.2° 2θ (blue arrows).

**Figure 4:**
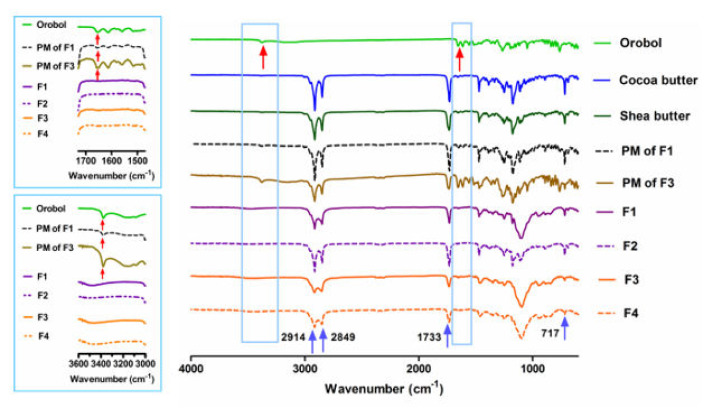
Fourier transform infrared (FTIR) analysis (relative intensity) of the orobol, solid lipids (i.e., cocoa butter and shea butter), PMs of F1 and F3, and lyophilized SLNs and NLCs formulations (F1 to F4). Distinctive bands of the orobol are presented at 1654 cm^−1^ and 3378 cm^−1^ (red arrows), while those of the lipids are presented at 717 cm^−1^, 1733 cm^−1^, 2849 cm^−1^, and 2914 cm^−1^ (blue arrows).

The pXRD patterns and ATR-FTIR analysis of the orobol, solid lipids (i.e., cocoa butter and shea butter), PMs of the F1 and F3 formulations (without surfactants), and the lyophilized SLNs and NLCs formulations (F1 to F4) are shown in Figure 3 and Figure 4, respectively. Crystalline patterns of the orobol and solid lipids are clearly observed. In the PM samples, the peaks of the orobol are still observed at 25.5° and 28.8° 2θ (red arrows, Figure 3), although their relative intensity are decreased. However, the orobol peaks are not observed in the lyophilized SLNs and NLCs formulations (F1 to F4), implying that the orobol was entrapped in the formulations in an amorphous and/or solubilized form. Similar to these results, distinctive bands of orobol at 1654 cm^−1^ and 3378 cm^−1^ can be observed in the intact powder and PMs (red arrows, Figure 4), but not in the prepared SLNs and NLCs formulations.

### 3.3. Stability of Orobol-Loaded SLNs and NLCs

**Figure 5:**
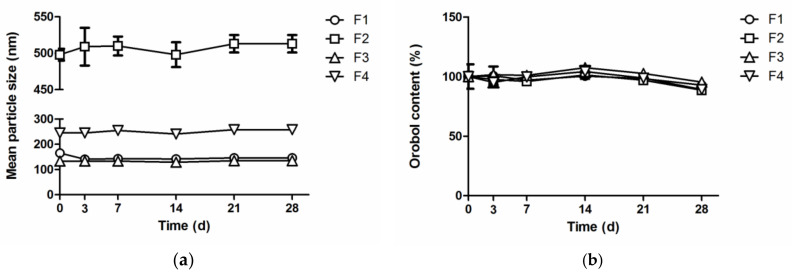
(**a**) The change of the mean particle size and (**b**) orobol content (% of initial day) in orobol-loaded formulations at room temperature under sunlight.

The stability of orobol in the F1–F4 formulations under sunlight was evaluated at room temperature by determining the change of mean particle size and orobol content (Figure 5). The mean particle size of the SLNs and NLCs formulations in aqueous solution (F1 to F4) was not significantly changed for 28 days, indicating that the aggregation of particles is negligible. Moreover, the orobol content (% of initial day) of the SLNs and NLCs formulations was also maintained up to 95.3% and 89.2%, respectively, for 28 days.

The color change of orobol in various vehicles and orobol-loaded formulations at room temperature with or without exposure to sunlight was observed for 14 days (Supplementary Figures S1 and S3). The color of the solutions containing orobol (0.05%, *w*/*w*) changed to yellow in the 14 days when exposed to sunlight. Although the ethanol solution containing orobol remained transparent when stored without sunlight, its color also changed to yellow when exposed to sunlight (Supplementary Figure S1). However, the SLNs and NLCs formulations (F1 to F4) maintained their initial white color for 14 days, regardless of exposure to sunlight, and an apparently yellow color was not notable (Supplementary Figure S3).

### 3.4. In Vitro Strat-M Membranes and Human Cadaver Skin Deposition of Orobol

**Figure 6:**
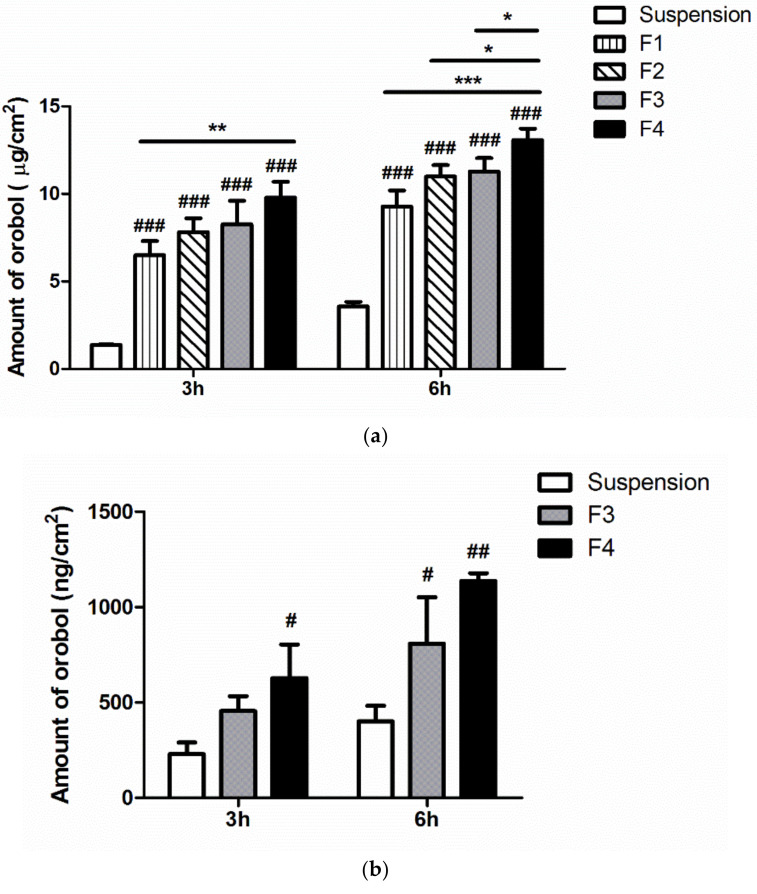
In vitro deposition of orobol into (**a**) Strat-M membranes and (**b**) human cadaver skin after applying the orobol-loaded SLNs and NLCs formulations (F1–F4) or orobol suspension in deionized water (DW) at a concentration of 0.5 mg/mL of orobol for 3 h and 6 h. Significantly different from one another at * *p* < 0.05, ** *p* < 0.01, and *** *p* < 0.001; Significantly different from the suspension group at # *p* < 0.05, ## *p* < 0.01, and ### *p* < 0.001.

The in vitro deposition of orobol into artificial membranes (Strat-M) and human cadaver skin after applying the orobol-loaded formulations or orobol suspension for 3 h and 6 h is shown in Figure 6. The amount of orobol deposited into the Strat-M membranes from the SLNs and NLCs formulations (F1 to F4) was significantly enhanced compared to suspension group at both 3 h and 6 h (*p* < 0.001). Notably, the deposited amount of orobol was the highest in F4 at both 3 h and 6 h among all of the tested samples, followed by F3, F2, and F1. Thus, the F3 and F4 formulations prepared using shea butter as a solid lipid were selected for further in vitro deposition study using human cadaver skin. Similar to the results using artificial membranes (Figure 6a), the deposited amount of orobol into human cadaver skin from F3 (*p* < 0.05) and F4 (*p* < 0.01) was significantly higher than that from the suspension group at 6 h, while only F4 group was significantly different from the suspension group at 3 h (*p* < 0.05) (Figure 6b). Moreover, the deposited amount of orobol was higher in F4 than in F3 at both 3 h and 6 h. Thus, F4 was selected as the optimum formulation for further in vivo skin irritation study, based on the results of the skin deposition studies.

### 3.5. Skin Irritation of Orobol-Loaded NLCs in Humans

After applying the blank NLCs formulation (blank F4) or the orobol-loaded NLCs formulation (F4) on the back of human volunteers for 24 h, the skin irritation scores and index determined at 30 min, 24 h, and 48 h are summarized in Supplementary Tables S3 and S4, respectively. Based on the classification system by the EPA (No irritancy: 0 ≤ irritation index < 0.2; Supplementary Table S2), it was confirmed that neither the blank NLCs nor the orobol-loaded NLCs formulation (irritation index: 0.0167) caused significant skin irritation in human skin (Table 4).

## 4. Discussion

In the present study, lipid nanoparticle formulations (SLNs and NLCs) were prepared to improve the stability and skin deposition of poorly water-soluble orobol. The melting point of cetyl palmitate and Compritol 888 ATO, frequently used in SLNs and NLCs formulations, is 54 °C and 71.2 °C, respectively. However, cocoa butter and shea butter were selected as the solid lipids in this study since their melting points (34.1 °C and 36.6 °C, respectively) are relatively close to the temperature of human skin. Moreover, both of the solid lipids would melt at body temperature after application, which is expected to help the absorption of orobol into the skin [27]. Capmul MCM EP was selected as an oil (liquid lipid) to prepare the orobol-loaded NLCs formulations, based on the solubility of orobol (Table 1). Surfactants also have a critical effect on the stability of lipid nanoparticle formulations; it has been reported that several surfactants reduce the interfacial tension between the solid lipid phase and aqueous phase, thereby enhancing the thermodynamic stability of SLNs and NLCs formulations [28]. In the current research, Transcutol was selected as the surfactant based on the solubility study (Table 1). Additionally, Tween 20 was included in the formulations, since it is known to be more effective in decreasing the surface tension of lipid nanoparticles than other non-ionic surfactants such as tween 80, thereby maintaining their stability [29].

The orobol-loaded formulations were characterized by their physicochemical properties, including their mean particle size, EE, loading content, size distribution, and particle morphology of (Table 3 and Figure 2). Notably, the mean particle size of the NLCs formulations (F2, 498 nm; F4, 246 nm) containing Capmul MCM EP as an oil was larger than that of the SLNs formulations (F1, 165 nm; F3, 133 nm). This increase in particle size is consistent with other previous studies that have reported larger sizes in NLCs formulations when oil is added into the solid lipid phase of SLNs [30,31,32]. However, the high EE (>95%) and orobol content (≈1.0%) in the F1–F4 formulations were not significantly affected by the existence of oil and an increase of particle size. The pXRD patterns were observed to determine changes in the crystallinity of orobol in the prepared formulations. As shown in Figure 3, when the ratio of orobol to shea butter in the PMs was increased to 1:2 (*w*/*w*) to verify the orobol crystalline peaks, they were more clearly observed at the same 2θ (red arrows, Figure 3), which confirms that the crystallinity of orobol was remained in the PMs. However, the distinctive pattern of orobol (crystalline form) disappeared in the orobol-loaded SLNs and NLCs formulations (F1 to F4), implying that orobol was solubilized and/or dispersed in an amorphous form in the lipid phase. In the ATR-FTIR analysis of orobol, the distinctive bands at 1654 cm^−1^ and 3378 cm^−1^ (red arrows, Figure 4) corresponded with the stretching vibrations of the carbonyl groups and hydroxyl groups, respectively [33,34]. Similar to the pXRD results, these bands were also observed in the PMs, but not in the F1–F4 formulations. It is interesting to note that the pXRD analysis of the solid lipids also showed crystalline patterns at 19.3°, 22.1°, and 24.2° 2θ (blue arrows, Figure 3). However, the solid lipids peaks were significantly weakened in the SLNs and NLCs. This result implies that the crystallinity of the solid lipids was attenuated during the preparation of the SLNs and NLCs formulations and/or the lyophilization process [35]. Consistently, the distinctive FTIR bands of the solid lipids at 717 cm^−1^, 1733 cm^−1^, 2849 cm^−1^, and 2914 cm^−1^ (blue arrows, Figure 4), which corresponded with the rocking motion of the C-H groups and the stretching vibrations of the C=O, O-CH_2_, and C-H groups, respectively [36,37], were diminished in the SLN and NLC formulations.

The stability of orobol in various formulations was evaluated by determining the change of the mean particle size and orobol content. Previous studies have demonstrated that various soy isoflavones, including daidzein, glycitein, and genistein, whose chemical structures are similar to orobol, are instable when exposed to UV radiation [38,39]. Our preliminary study also consistently showed the color change of orobol in various solutions when exposed to sunlight (Supplementary Figure S1). However, apparent color change was not observed in the SLNs and NLCs formulations for up to 14 days (Supplementary Figure S3). Moreover, more than 90% of the orobol content was maintained in the SLNs and NLCs formulations for up to 28 days under sunlight (Figure 5), while only 76.1 ± 0.8% of the orobol content remained in methanol solution after seven days with exposure to sunlight (data not shown). Furthermore, the SLNs and NLCs formulations did not aggregate during storage, and maintained their initial particle size for up to 28 days. This improvement in the stability may be attributed to the entrapment of orobol into the inner phase of the SLNs and NLCs formulations comprising solid lipid and liquid lipid. We assume that the orobol entrapped into the inner lipids could have less interaction with the outer aqueous phase and thus are more protected from sunlight, thereby being degraded more slowly. Moreover, the constituents of shea butter, such as tocopherol and phenolic compounds, could prevent orobol from oxidation due to their anti-oxidant properties [35]. Thus, it can be confirmed that the stability of orobol in the prepared lipid nanoparticles (SLNs and NLCs) could be significantly enhanced for up to 28 days.

In vitro deposition studies were conducted using Strat-M membranes and female human cadaver skin. Strat-M membranes that mimic intact full-thickness skin and act as penetration barriers have previously been used to determine the skin permeation and deposition of various compounds, as an alternative to animal and human skin [23,40,41]. As shown in Figure 6a, the SLNs and NLCs formulations showed a significantly higher deposition of orobol into the Strat-M membranes, compared to the suspension. Based on the Fick’s diffusion law, the increased solubility of amorphous orobol in the SLNs and NLCs formulations (Figure 3) could enhance the driving force of diffusion (by a high-concentration gradient) through the membranes, resulting in a higher deposition of orobol [16]. Surfactants and oil in the formulations also could act as a penetration enhancer [16,42]. The deposited amount of orobol into human cadaver skin was also significantly higher when the SLNs or NLCs formulations was applied, compared with the suspension (Figure 6b). In previous studies, it was demonstrated that the skin hydration effect by the occlusive properties of SLNs and NLCs formulations is higher than that of conventional formulations including suspension. Thus, they can promote skin penetration and retention more deeply into the skin [18,43]. It is interesting to note that using shea butter in the formulations resulted in higher deposition of orobol into the Strat-M membranes compared to cocoa butter (Figure 6a). Although the exact reason is unclear, the smaller particle size of shear butter formulations (F3 and F4) compared to that of cocoa butter (F1 and F2) could be one explanation, which needs further systematic studies in relation to trans-epidermal water loss (TEWL). Notably, the deposited amount of orobol into the Strat-M membranes and human cadaver skin was higher from the NLCs formulations than that from the SLNs formulations, which might be caused by the penetration-enhancing effect of the oil added into the solid lipid of the NLCs formulations [16]. Assuming that the epidermis/dermis layer of human skin is 1–2 mm thick [16,44], the concentration of orobol in the target human skin layer was calculated to be 11.0–19.9 μM from F4 in Figure 6b. Since orobol showed a potent anti-skin-aging effect via the inhibition of MMP-1 expression at a concentration range of 4–8 μM [15], it can be claimed that the prepared NLCs formulation can provide sufficient epidermal/dermal orobol concentration to exert its anti-skin-aging effect. Although the content of orobol in NLCs formulations are very low (<1%), the loaded orobol can be absorbed into the target region of the skin at an effective concentration. Therefore, the NLCs formulation containing shea butter (F4) was selected as the optimal formulation among those tested for further skin irritation study in humans. The final NLCs formulation with and without orobol (F4 and blank F4) did not cause skin irritation in the human study (Table 4), which implies that the constituents of the NLCs formulation have good biocompatibility after topical application.

In our preliminary studies, orobol-loaded oil-in-water microemulsion (o/w ME) formulation as a conventional colloidal carrier was also prepared for comparison. The prepared o/w ME formulation (Supplementary Table S4) was observed as nano-sized (253 nm) spherical droplets (Supplementary Figure S4). However, orobol-loaded o/w ME formulation did not improve the stability of orobol, since the particle size increased with the decrease of the orobol content during the storage for 28 days (Supplementary Figure S5). These results are consistent with a previous study that reported improved stability of tretinoin in SLNs and NLCs formulations compared to other lipid nano-vesicular systems [45]. Moreover, the orobol deposition from o/w ME formulation into Strat-M membranes was not significantly different with suspension group (Supplementary Figure S6). Those deposition results (NLCs > SLNs > Suspension ≈ *o*/*w* ME) were consistent with a previous study that investigated a topical psoralen delivery using NLCs compared with SLNs and suspension [46]. Thus, the NLCs formulation could be a promising delivery system for enhancing the stability and topical skin application of orobol.

## 5. Conclusions

The NLCs formulation containing shea butter was successfully optimized for improving the stability of orobol and enhancing its in vitro topical skin delivery. The NLCs significantly enhanced the deposition of orobol into the Strat-M membrane and human cadaver skin. Moreover, the NLCs formulation did not cause any skin irritation in the human study. Thus, the shea butter-based NLCs formulation prepared in this study could be a promising topical skin delivery system of orobol for enhancing the anti-skin-aging effect without skin irritation.

## Figures and Tables

**Table 1:** Solubility of orobol in various vehicles.

Phase	Vehicle	Solubility (mg/mL)
Water	DW	0.04 ± 0.01
Oil	Capmul MCM EP	12.37 ± 0.12
	Miglyol	1.03 ± 0.03
	MCT	1.03 ± 0.05
	Larbrafac CC	0.89 ± 0.07
Surfactant	Transcutol	67.94 ± 1.98
	Labrasol	54.53 ± 3.62
	Tween 20	23.81 ± 0.80

**Table 2:** Composition of solid lipid nanoparticle (SLNs) and nanostructured lipid carrier (NLCs) formulations (%, *w*/*w*) containing 0.05% (*w*/*w*) of orobol.

Phase	Vehicle	F1	F2	F3	F4
Solid lipid	Cocoa butter	1.5	1.5	-	-
	Shea butter	-	-	1.5	1.5
Oil	Capmul MCM EP	-	0.3	-	0.3
Surfactant	Transcutol	2	2	2	2
	Tween 20	2	2	2	2
Water	DW	94.45	94.15	94.45	94.15

**Table 3:** Physicochemical properties of the orobol-loaded NLCs and SLNs.

**Formulation**	**Particle Size (nm)**	**Polydispersity Index (PDI)**	**Entrapment Efficiency (EE, %)**	**Loading Content (%)**
F1	165 ± 3	0.211 ± 0.001	95.7 ± 2.5	0.96 ± 0.09
F2	498 ± 8	0.166 ± 0.042	95.9 ± 10.5	0.97 ± 0.07
F3	133 ± 6	0.140 ± 0.016	97.2 ± 4.1	0.93 ± 0.04
F4	246 ± 9	0.196 ± 0.016	96.8 ± 2.1	0.91 ± 0.04

**Table 4:** Skin irritation index of the blank NLC formulation (blank F4) and the orobol-loaded NLC formulation (F4) at 30 min, 24 h, and 48 h after the topical application to human volunteers for 24 h.

Formulation	30 Min	24 H	48 H
not-treated	0	0	0
blank F4	0.0167	0.0167	0.0167
F4	0.0167	0.0167	0.0167

## Supplementary Materials

The following are available online at https://www.mdpi.com/1999-4923/12/9/845/s1, Figure S1: The color change of orobol (0.05%, *w*/*w*) in various vehicles. (a) Samples immediately after the preparation; (b) samples stored at room temperature for 14 days without sunlight exposure; (c) samples stored at room temperature for 14 days with sunlight exposure, Figure S2: (a) The LC-MS/MS peak of orobol at a concentration of the lower limit of quantification (10.0 ng/mL); (b) the calibration standard curve of orobol at a final concentration range of 10.0 –1000 ng/mL, Figure S3: The color change of the orobol-loaded formulations. (a) Samples immediately after the preparation; (b) samples stored at room temperature for 14 days without exposure to sunlight; (c) samples stored at room temperature for 14 days with exposure to sunlight, Figure S4: (a) Morphological shapes of o/w ME formulation by TEM; (b) size distribution of o/w ME formulation. The scale bars represent 0.5 μm, Figure S5: The change of mean particle size (left *Y*-axis; ●) and content (% of initial concentration; right *Y*-axis; ○) in o/w ME formulation at room temperature on day 0 (initial state after the preparation), day 3, 7, 14, 21, and 28, Figure S6: In vitro deposition of orobol into a Strat-M membrane after applying orobol-loaded o/w ME formulation at the concentration of 0.5 mg/mL for 3 h and 6 h, Table S1: Criteria of the skin irritation score system by the guidelines of the International Contact Dermatitis Research Group (ICDRG) and the Personal Care Products Council (PCPC), Table S2: The standard procedure dermal classification system by the Environmental Protection Agency (EPA), Table S3: Skin irritation scores of the blank nanostructured lipid carrier (NLC) formulation (blank F4) and the orobol-loaded NLC formulation (F4) at 30 min, 24 h, and 48 h after topical application of the samples to human volunteers for 24 h, Table S4: Composition of o/w ME formulation (w/w, %) containing 0.05(w/w, %) of orobol.
