# Supplementary material for: Lipid Nanoparticles for Enhancing the Physicochemical Stability and Topical Skin Delivery of Orobol
Source: Pharmaceutics. 2020 Sep 3;12(9):845. doi: 10.3390/pharmaceutics12090845 (PMC7560103; doi:10.3390/pharmaceutics12090845)
Supplement: Supplementary file 1 [file pharmaceutics-12-00845-s001.pdf]

pharmaceutics

MDPI

Article

Supplementary Materials: Lipid Nanoparticles for Enhancing the Physicochemical Stability and Topical Skin Delivery of Orobol

Min-Hwan Kim, Yae-Eun Jeon, Soobeen Kang, Jae-Young Lee, Ki Won Lee, Ki-Taek Kim and Dae-Duk Kim

(a)

(b)

(c)

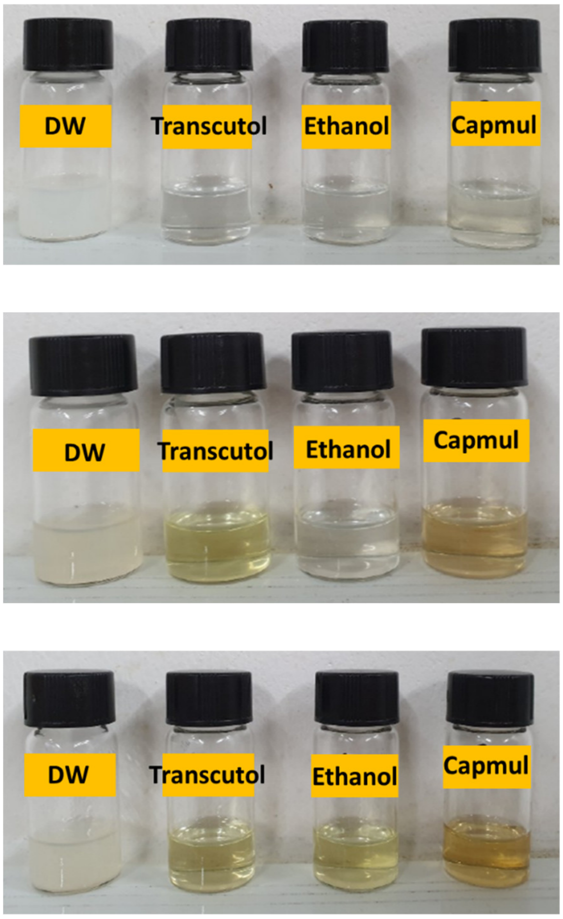

Figure S1. The color change of orobol (0.05%, *w*/*w*) in various vehicles. (**a**) Samples immediately after the preparation; (**b**) samples stored at room temperature for 14 days without sunlight exposure; (**c**) samples stored at room temperature for 14 days with sunlight exposure.

(a)

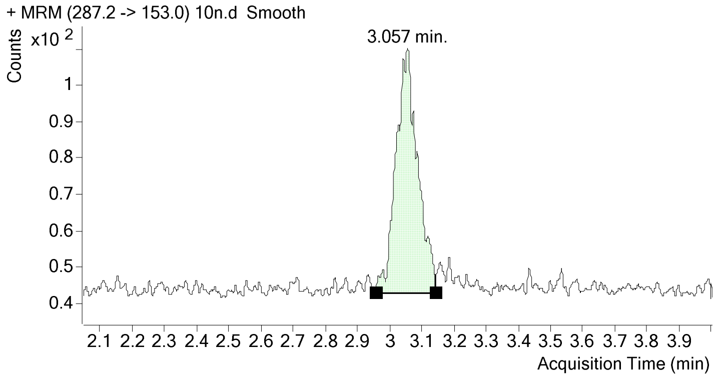

(b)

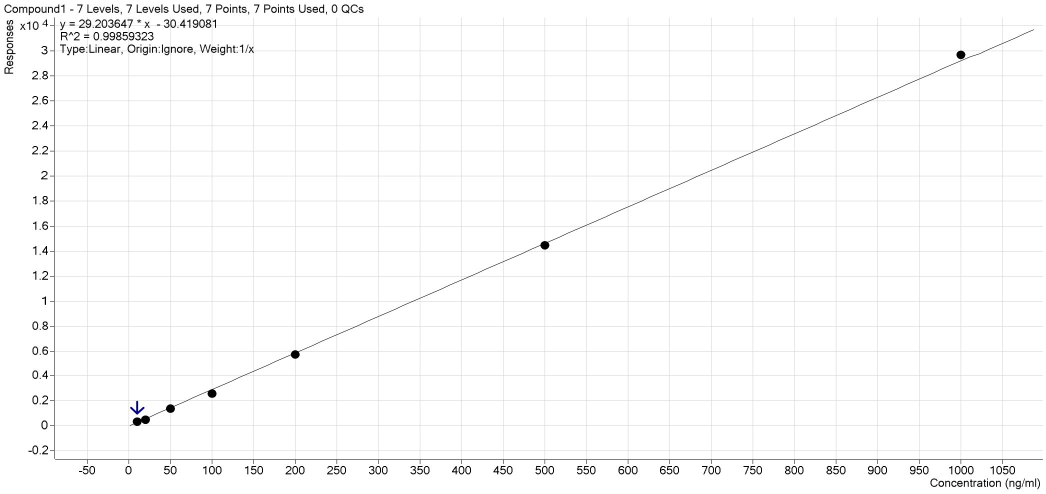

Figure S2. (**a**) The LC-MS/MS peak of orobol at a concentration of the lower limit of quantification (10.0 ng/mL); (**b**) The calibration curve of orobol at a final concentration range of 10.0 – 1000 ng/mL.

(a)

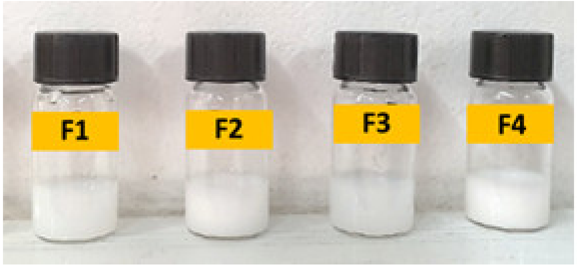

(b)

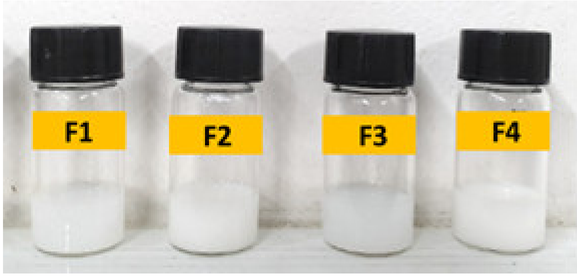

(c)

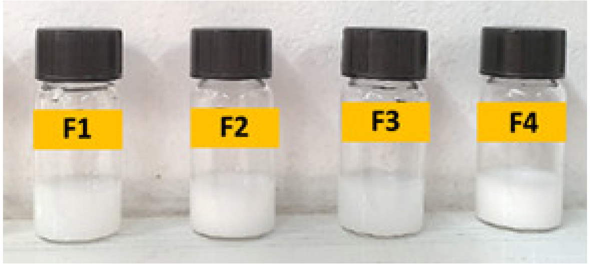

Figure S3. The color change of the orobol-loaded formulations. (**a**) Samples immediately after the preparation; (**b**) Samples stored at room temperature for 14 days without exposure to sunlight; (**c**) Samples stored at room temperature for 14 days with exposure to sunlight.

(a)

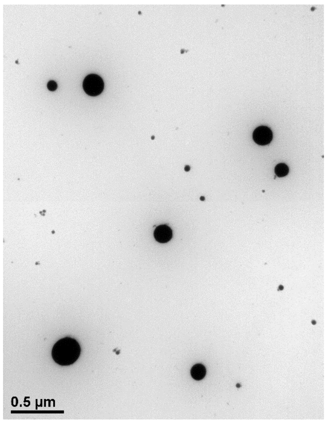

(b)

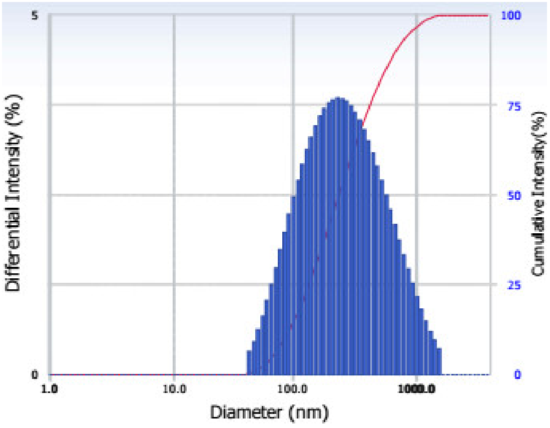

Figure S4. (**a**) Morphological shapes of *o*/*w* ME formulation by TEM and (**b**) its size distribution. The scale bars represent 0.5 µm.

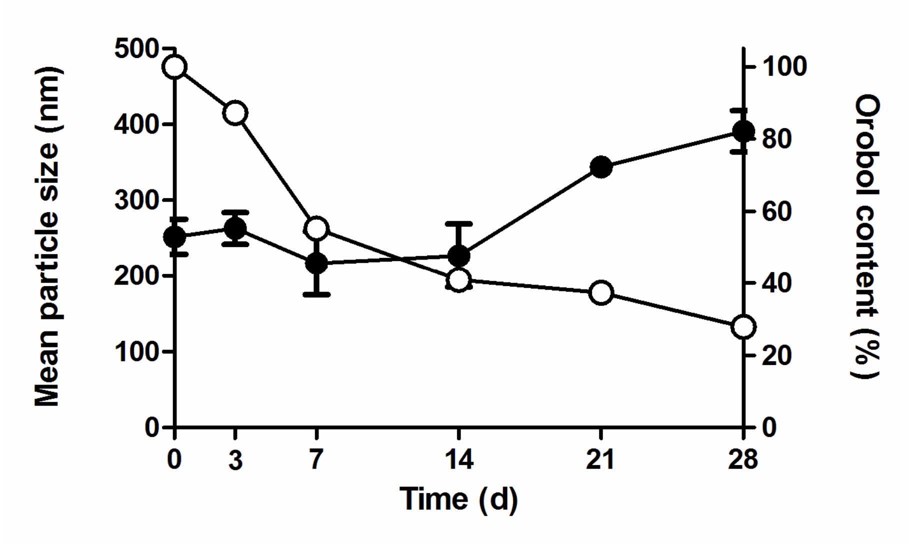

Figure S5. The change of mean particle size (left Y-axis; •) and content (% of initial concentration; right Y-axis; ○) in *o/w* ME formulation at room temperature on day 0 (initial state after the preparation), day 3, 7, 14, 21, and 28.

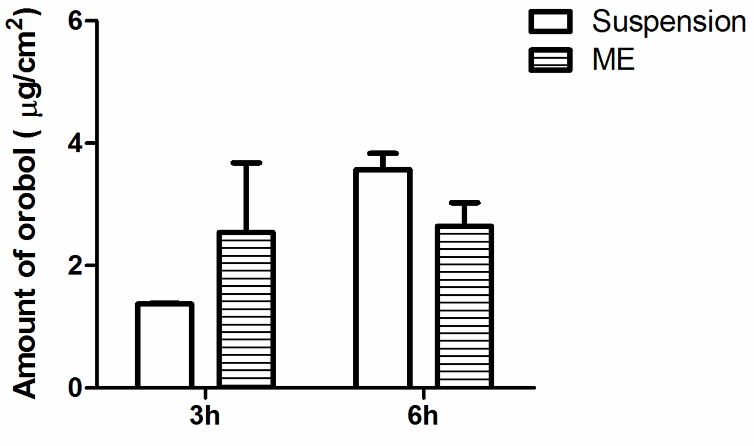

Figure S6. In vitro deposition of orobol into a Strat-M membrane after applying orobol-loaded *o/w* ME formulation at the concentration of 0.5 mg/mL for 3 h and 6 h.

Table S1. Criteria of the skin irritation score system by the guideline of the International Contact Dermatitis Research Group (ICDRG) and the Personal Care Products Council (PCPC).

Score	Criteria for Skin Irritation
0	No signs of irritation
0.5	Doubtful or slight reaction
1	Slight erythema
2	Moderate erythema with or without partial edema
3	Moderate erythema with diffusive edema
4	Intense erythema with diffusive edema containing vesicles

Table S2. The standard procedure dermal classification system by the Environmental Protection Agency (EPA).

Skin Irritation Index	Classification of Skin Irritation
0.00–0.02	No irritancy
0.02–0.25	Low irritancy
0.25–1.00	Slight irritancy
1.00–2.50	Moderate irritancy
>2.50	Severe irritancy

Table S3. Skin irritation scores of the blank nanostructured lipid carrier (NLC) formulation (blank F4) and the orobol-loaded NLC formulation (F4) at 30 min, 24 h, and 48 h after topical application of the samples to human volunteers for 24 h.

Human Subject	Not-Treated			Blank F4			F4		
	30 min	24 h	48 h	30 min	24 h	48 h	30 min	24 h	48 h
No. 1	0	0	0	0	0	0	0	0	0
No. 2	0	0	0	0	0	0	0	0	0
No. 3	0	0	0	0	0	0	0	0	0
No. 4	0	0	0	0	0	0	0	0	0
No. 5	0	0	0	0	0	0	0	0	0
No. 6	0	0	0	0	0	0	0	0	0
No. 7	0	0	0	0	0	0	0	0	0
No. 8	0	0	0	0	0	0	0	0	0
No. 9	0	0	0	0	0	0	0	0	0
No. 10	0	0	0	0	0	0	0	0	0
No. 11	0	0	0	0	0	0	0	0	0
No. 12	0	0	0	0	0	0	0	0	0
No. 13	0	0	0	0	0	0	0	0	0
No. 14	0	0	0	0.5	0.5	0.5	0.5	0.5	0.5
No. 15	0	0	0	0	0	0	0	0	0
No. 16	0	0	0	0	0	0	0	0	0
No. 17	0	0	0	0	0	0	0	0	0
No. 18	0	0	0	0	0	0	0	0	0
No. 19	0	0	0	0	0	0	0	0	0
No. 20	0	0	0	0	0	0	0	0	0
No. 21	0	0	0	0	0	0	0	0	0
No. 22	0	0	0	0	0	0	0	0	0
No. 23	0	0	0	0	0	0	0	0	0
No. 24	0	0	0	0	0	0	0	0	0
No. 25	0	0	0	0	0	0	0	0	0
No. 26	0	0	0	0	0	0	0	0	0
No. 27	0	0	0	0	0	0	0	0	0
No. 28	0	0	0	0	0	0	0	0	0
No. 29	0	0	0	0	0	0	0	0	0
No. 30	0	0	0	0	0	0	0	0	0

Table S4. Composition of o/w ME formulation (w/w %) containing 0.05(w/w %) of orobol.

Phase	Vehicle	ME Formulation
Oil	Capmul MCM EP	20
Surfactant	Transcutol	28.7
	Labrasol	14.3
Water	DW	36.95
